# MiR-34a regulates the invasive capacity of canine osteosarcoma cell lines

**DOI:** 10.1371/journal.pone.0190086

**Published:** 2018-01-02

**Authors:** Cecilia M. Lopez, Peter Y. Yu, Xiaoli Zhang, Ayse Selen Yilmaz, Cheryl A. London, Joelle M. Fenger

**Affiliations:** 1 Department of Veterinary Clinical Sciences, College of Veterinary Medicine, The Ohio State University, Columbus, Ohio, United States of America; 2 Medical Student Research Program, The Ohio State University College of Medicine, The Ohio State University Wexner Medical Center, Columbus, Ohio, United States of America; 3 Center for Biostatistics, Department of Biomedical Informatics, The Ohio State University, Columbus, Ohio, United States of America; 4 Department of Veterinary Biosciences, College of Veterinary Medicine, The Ohio State University, Columbus, Ohio, United States of America; 5 Department of Veterinary Biosciences, College of Veterinary Medicine, Tufts University, New Grafton, Massachusetts, United States of America; Colorado State University, UNITED STATES

## Abstract

**Background:**

Osteosarcoma (OSA) is the most common bone tumor in children and dogs; however, no substantial improvement in clinical outcome has occurred in either species over the past 30 years. MicroRNAs (miRNAs) are small non-coding RNAs that regulate gene expression and play a fundamental role in cancer. The purpose of this study was to investigate the potential contribution of miR-34a loss to the biology of canine OSA, a well-established spontaneous model of the human disease.

**Methodology and principal findings:**

RT-qPCR demonstrated that miR-34a expression levels were significantly reduced in primary canine OSA tumors and canine OSA cell lines as compared to normal canine osteoblasts. In canine OSA cell lines stably transduced with empty vector or pre-miR-34a lentiviral constructs, overexpression of miR-34a inhibited cellular invasion and migration but had no effect on cell proliferation or cell cycle distribution. Transcriptional profiling of canine OSA8 cells possessing enforced miR-34a expression demonstrated dysregulation of numerous genes, including significant down-regulation of multiple putative targets of miR-34a. Moreover, gene ontology analysis of down-regulated miR-34a target genes showed enrichment of several biological processes related to cell invasion and motility. Lastly, we validated changes in miR-34a putative target gene expression, including decreased expression of KLF4, SEM3A, and VEGFA transcripts in canine OSA cells overexpressing miR-34a and identified KLF4 and VEGFA as direct target genes of miR-34a. Concordant with these data, primary canine OSA tumor tissues demonstrated increased expression levels of putative miR-34a target genes.

**Conclusions:**

These data demonstrate that miR-34a contributes to invasion and migration in canine OSA cells and suggest that loss of miR-34a may promote a pattern of gene expression contributing to the metastatic phenotype in canine OSA.

## Introduction

Osteosarcoma (OSA) is the most common form of malignant bone cancer in dogs and children, although the incidence of disease in the canine population is approximately ten times higher than that in people [1–3]. Both clinical and molecular evidence suggest that canine OSA exhibits a similar biology to its human counterpart including anatomic location, presence of early microscopic metastatic disease at diagnosis, development of chemotherapy-resistant metastases, altered expression/activation of several proteins (e.g. Met, PTEN, STAT3), and p53 inactivation, among others [2, 4]. Additionally, canine and pediatric OSA exhibit overlapping transcriptional profiles and shared DNA copy number aberrations, supporting the notion that these diseases possess significant similarity at the molecular level [5–8]. Indeed, canine OSA has been used as a spontaneous large animal model of the human disease to study OSA biology and investigate the clinical efficacy of novel therapeutic approaches such as limb-sparing surgery, immunotherapy treatments, and aerosolized chemotherapy delivery [9–12]. While the adoption of multidrug chemotherapy protocols and aggressive surgical techniques has improved survival, approximately 30% of children and over 90% of dogs ultimately die of disease and no substantial improvement in clinical outcome has occurred in either species over the past 30 years.

MicroRNAs (miRNAs) are small noncoding RNAs that regulate gene expression at the post-transcriptional level through either mRNA cleavage and/or translational repression. Their functions extend to both physiological and pathological conditions, including cell fate specification, cell death, development, metabolism, and cancer [13, 14]. Accumulating evidence suggests that miRNAs can function as either tumor suppressors or oncogenes by targeting genes involved in tumor development and progression in a variety of cancers, making them relevant targets for therapeutic intervention [15–19]. In support of this, chemically modified oligonucleotides can downregulate the expression and the function of miRNAs in malignant cells thereby altering cancer phenotypes *in vitro* [20–24].

Among the miRNAs implicated in cancer development and progression, the miR-34 family has been intensively studied and data indicate family members function as tumor suppressors in a variety of human cancers [25, 26]. The miR-34 family consists of three evolutionarily conserved miRNAs: MiR-34a, MiR-34b and MiR-34c. The mature miR-34a sequence is located within the second exon of its non-coding host gene whereas miR-34b and miR-34c are co-transcribed and located within a single non-coding precursor (miR-34b/c) [25]. Deletions of the gene regions harboring these transcripts or CpG promoter methylation with miR-34 gene silencing are frequently observed in human malignancies including neuroblastoma, glioma, breast cancer, non-small cell lung cancer, colorectal cancer, and osteosarcoma [27–32]. Furthermore, p53-mediated transcriptional regulation of the miR-34 family is conserved across different cell types [33–37]. Indeed, the miR-34 family members function as tumor suppressors, inducing apoptosis, cell cycle arrest and senescence, in part, through their interaction with the p53 tumor suppressor network [33, 37–39]. For example, recent studies demonstrated that miR-34a contributes to cell cycle arrest and apoptosis through its repression of several p53 target genes, including CDK4, CDK6, and Bcl-2 [32, 40, 41].

With respect to OSA, miR-34a expression is decreased in human primary OSA tumor samples when compared to adjacent normal tissues, suggesting a role for miR-34a dysregulation in disease pathogenesis [30]. MiR-34 genes exhibited minimal deletions, loss of heterozygosity (LOH), and epigenetic inactivation in human OSA tumor tissues, demonstrating that other genetic and epigenetic mechanisms may account for the observed decrease expression [30]. Furthermore, restoration of miR-34a in human OSA cell lines reduced cell proliferation and migration *in vitro* and attenuated OSA tumor xenograft growth and metastasis *in vivo*, in part through targeting of the receptor tyrosine kinase Met [42]. Effects of miR-34 on OSA cell line migration and invasiveness appear to be at least partially mediated through repression of CD44, the receptor for hyaluronic acid and a well-established marker of cancer cell stemness [43]. In concordance with the potential role of miR-34a in malignant osteoblast behavior, decreased expression of miR-34a in spontaneous human OSA tumors and low levels of circulating miR-34a in OSA patient plasma are associated with shorter disease-free survival and poor prognosis [44, 45].

Studies evaluating miRNA expression in spontaneously occurring canine OSA demonstrate that similar to human cancers, alteration of miRNAs are consistently observed. Our laboratory has recently identified a unique miRNA expression signature associated with primary canine OSA tumors that is distinct from that of normal canine osteoblasts [46]. Significantly, several members of this miRNA signature are known to be aberrantly expressed in human OSA tumors, including miR-34a which is also downregulated in canine OSA. The purpose of this study was to investigate the potential contribution of miR-34a loss to the biology of canine OSA, a well-established spontaneous model of the human disease, and evaluate the functional consequences of altered miR-34a expression in canine OSA cell lines.

## Methods

### Cell lines and primary tumor samples

Tumor sample collections from client-owned dogs were performed in compliance with established hospital protocols and approved by the Institutional Animal Care and Use Committee (IACUC #2010A0015) at The Ohio State University Veterinary Medical Center (OSU CVM). Informed consent was obtained from all owners prior to all sample collection. Canine OSA tumor tissues were obtained from dogs treated at the OSU CVM and all tumors were evaluated and confirmed as OSA by board certified veterinary pathologists at the OSU CVM. Clinical patient data, including age, sex, breed, histopathological diagnosis, and primary tumor location is detailed in S1 Table. Canine OSA cell lines OSA2, OSA8, OSA16, OSA40, and OSA50 were kindly provided by Dr. Jaime Modiano (University of Minnesota, Minneapolis, MN, USA). The canine Abrams cell line was kindly provided Dr. Doug Thamm (Colorado State University, Fort Collins, CO, USA). The canine D17 cell line was purchased from ATCC (Cat. # ATCC® CCL-183). Canine osteoblast cells were purchased from Cell Applications (Cell Applications Inc.) and cultured in Canine Osteoblast Growth Medium (Cat. # Cn417-500, Cell Applications Inc.) according to manufacturer’s instructions. The canine OSA8 and Abrams cell lines were maintained in RPMI-1640 (Gibco Life Technologies, Grand Island, NY, USA) or DMEM (Gibco Life Technologies) supplemented with 10% fetal bovine serum, non-essential amino acids, sodium pyruvate, penicillin, streptomycin, L-glutamine, and HEPES (4-(2-dydroxethyl)-1-piperazineethanesulfonic acid) at 37°C, supplemented with 5% CO_2_ (media supplements from Gibco).

### RNA isolation, cDNA synthesis, and RT-qPCR

RNA was extracted from OSA cell lines and primary OSA tumors using the RNeasy Mini Kit (QIAGEN, Hilden, Germany) according to the manufacturer’s instructions. RT-qPCR was performed using the Applied Biosystems StepOne Plus Detection System (Applied Biosystems, Foster City, CA, USA). Human Taqman miRNA assays (Applied Biosystems) were used according to manufacturer’s instructions to quantify mature miR-34a expression in canine cell lines and tissues (mature miR-34a shares 100% sequence homology between dogs and humans) [46]. 50 ng total RNA was converted to first-strand cDNA with miRNA-specific primers, followed by RT-qPCR with TaqMan probes. The “NormFinder” algorithm was used to analyze the expression stability/variation value among a set of candidate genes and identify the optimal normalization gene [47]. The expression stability (V) for three endogenous controls (U6 snRNA, RNU44, and RNU48) was determined and snRNA U6 (V = 0.359) was ranked as the most stable gene expressed in OSA cell lines and primary OSA tissues as compared to RNU44 (V = 0.536) or RNU48 (V = 2.591) and therefore selected as the normalizer gene for all miRNA-specific RT-qPCRs. RT-qPCR was performed to validate changes in mRNA expression for selected genes affected by miR-34a over expression. cDNA was made from 1 μg of total RNA using Superscript III (Invitrogen). KLF4, SEMA3E, VEGFA, and GAPDH transcripts were detected using Fast SYBR green PCR master mix (Applied Biosystems) according to the manufacturer’s protocol. Primers for canine KLF4, SEMA3E, VEGFA, and GAPDH were designed and primer efficiency was determined by constructing serial dilution curves and calculating the coefficient of determination (R^2^) and amplification efficiency for each primer set. All primers demonstrated high amplification efficiency (94–98%) with a correlation coefficient (R^2^) ≥ 0.95 for all primer sets; primers are detailed in Table 1. NormFinder analysis found that canine GAPDH had the lowest stability value (V = 0.048) in both OSA cell lines and primary OSA tumors compared to 18S (V = 0.116) and β-Actin (V = 0.350) candidate normalization genes. GAPDH represented the most stable housekeeping gene; therefore, normalization was performed relative to GAPDH internal control. All reactions were performed in triplicate and included no-template controls for each gene. Relative gene expression for all RT-qPCR data was calculated using the comparative threshold cycle method [48].

Experiments were repeated 3 times using samples in triplicate.

**Table 1:** List of reference genes and target genes used for RT-qPCR.

Gene/primers	Primer sequences	Primer efficiency (%)	Correlation coefficient (R^2^)
Canine RELN 1354F Canine RELN 1578R	5’GCA TGA AGC TCG AGT CCA CGC ACT CG 3’ 5’CCG CAG TTC ACG CAC TCC CTG C 3’	94.6	0.98
Canine KLF4 457F Canine KLF4 606R	5’GCC ACC TGG CGA GTC TGA CAT G 3’ 5’CGC TTC ATG TGC GAG AGC TCC TC 3’	97.1	0.95
Canine SEMA3E 766F Canine SEMA3E 935R	5’GCT CTG TAC CAG GAA TGA ATG 3’ 5’CCG GAT GCT AGA CAT ATG ATA C 3’	95.4	0.97
Canine VEGFA 299F Canine VEGFA 429R	5’GTG CAT TGG AGC CTT GCC TTG CTG 3’ 5’CAG TAG CTG CGC TGG TAG ACG TC 3’	98.1	0.97
Canine GAPDH F Canine GAPDH R	5’GTC CAT GCC ATC ACT GCC ACC CAG 3’ 5’CTG ATA CAT TGG GGG TGG GGA CAC 3’	97.4	0.96

### miR-34a lentivirus infection

Lentiviral constructs were purchased from Systems Biosciences (Mountain View, CA, USA). Packaging of the lentiviral constructs was performed using the pPACKH1 Lentivector Packaging KIT (catalog no. LV500A-1) according to the manufacturer’s instructions. Canine OSA8 and Abrams cells were transduced with empty lentivirus (catalog no. CD511B-1) or pre-miR-34a lentivirus (catalog no. PMIRH34aPA-1). Briefly, 5 x 10^5^ cells were plated and left overnight in 10% serum-containing medium. The following day, the medium was changed to Stemline (Gibco) with transfection agent TransDux (Systems Biosciences) and either pre-miR-34a or control empty virus was added to cells overnight according to manufacturer’s protocol. Cells were FACS sorted based on GFP positivity 72 hour post-transduction and cells expressing the highest mean fluorescent intensity (top 30% of GFP+ population) were collected. Cells were expanded in culture for 5–7 days and miR-34a expression was determined by RT-qPCR (Applied Biosystems). After confirming overexpression of miR-34a lentiviral constructs, cells were plated immediately for functional assays (cell proliferation, cell cycle analysis, invasion/migration).

### Assessment of cell proliferation

Changes in cell proliferation were assessed using a commercially available bromodeoxyuridine (BrdU) incorporation assay (Roche, Basel, CH). Briefly, 2 x 10^3^ Abrams or OSA8 cells transduced with either pre-miR-34a or negative control empty virus were plated in complete medium for 24, 48, or 72 hours in 96-well plates. The BrdU reagent was added, cells were incubated for another 3 hours and then harvested, fixed, and digested by nuclease for 30 minutes at 37°C. Cells were then incubated with conjugate for 1 hour and washed, and the plates were developed by adding 100 μL of substrate for 30 minutes. The absorbance was measured using an enzyme-linked immunosorbent assay (ELISA) plate reader (Spectra Max; Molecular Devices, Sunnyvale, CA, USA). Experiments were repeated 3 times for each cell line.

### Cell-cycle analysis

The propidium iodide staining method was used to assess the effects of miR-34a on cell cycle distribution [49]. In brief, OSA8 or Abrams cells (5.0 x 10^6^ cells/well) transduced with pre-miR-34a or empty vector control lentivirus were seeded in 6-well plates in 3 mL of RPMI-1640 (OSA8) or DMEM (Abrams) with 10% fetal bovine serum and incubated overnight at 37°C and 5% CO2. Cells were then collected, washed in 0.1% glucose/PBS, and then fixed in cold 70% ethanol at 4° overnight. Cells were incubated with 200 μL of propidium iodide working solution consisting of propidium iodide (50 μg/mL) and RNAse (10 μg/mL) in PBS containing 0.1% glucose, and analyzed by means of flow cytometry (BD Accuri C6, BD Biociences, San Jose, CA, USA). Data were analyzed by means of standard software (BD Accuri™ C6 Software). Each experiment was repeated 3 times with each cell line.

### Matrigel invasion assay

A Matrigel invasion assay was performed to evaluate the impact of miR-34a on invasion. Cell culture inserts (8-μm pore size; Falcon) were coated with 100 μL of diluted (1:10) Matrigel (BD Bioscience, San Jose, CA, USA) and allowed to solidify at 37°C for 1 h to form a thin continuous layer. Canine OSA8 (5 x 10^4^) or Abrams cells (1 × 10^5^) transduced with empty vector or pre-miR-34a lentivirus were prepared in serum-free medium and seeded into each insert (upper chamber) and media containing 10% fetal bovine serum was placed in the lower chamber. Cells were incubated for 24 hours to permit invasion through the Matrigel layer. Inserts were processed and cells counted as previously described [46]. Samples were run in triplicate and cells from ten independent 20x high powered fields from each replicate were counted. Experiments were repeated 3 times for each cell line.

### Wound healing assay

To evaluate the effects of miR-34a on cell migration, 5 x 10^6^ canine OSA8 or Abrams cells transduced with control lentivirus or pre-miR-34a lentivirus were seeded in complete medium and grown until confluent in 6-well plates. A gap was introduced in the cells by scraping with a P200 pipette tip and cells were placed in fresh medium containing 10% fetal bovine serum. After 24 hours, cells were stained with crystal violet stain and migration across the gap was evaluated by digital photography. Each experiment was repeated 3 times.

### RNA sequencing

Total RNA was extracted from OSA8 cells transduced with either empty lentivirus (n = 3) or pre-miR-34a lentivirus (n = 3) using the RNeasy Mini Kit (QIAGEN) and RNA sequencing was performed at the OSU Comprehensive Cancer Center Genomics Shared Resource as previously described [46]. Total RNA was subject to ribosomal reduction and RNA-seq libraries were constructed using TruSeq stranded total RNA (Illumina; RS-122-2201) according to the manufacturer’s instructions. Sequencing was performed on an Illumina HiSEq. 2500 instrument at a depth of ∼40 million paired-end, 50 bp long, strand-specific reads per sample. AdapterRemover was used to trim adapter sequences and the remaining reads were aligned using STAR to the canFam3 genome. Quality control was performed using RNA-SeQC, to identify samples requiring additional sequencing reads. Differential expression was determined using DESeq2, which compared sample groups using a likelihood-ratio test (LRT). Data were quantile normalized and linear regressions were used to compare mRNA expression between samples. Differential gene expression was determined by one-way analysis of variance (ANOVA) and *p*-values of less than 0.05 were considered statistically significant.

Prediction of miR-34a binding to the 3’-UTR of genes down-regulated by miR-34a was performed with computer-aided algorithms obtained from TargetScan (http://www.targetscan.org), PicTar (http://pictar.mdc-berlin.de), miRanda (http://www.microrna.org), and miRWalk (http://www.umm.uni-heidelberg.de/apps/zmf/mirwalk). Down-regulated genes (fold change ≤ -1.4) that contained consensus binding sites for miR-34a in their 3’UTR were identified and gene ontology enrichment analysis was performed using the ToppGene database (https://toppgene.cchmc.org; FDR correction and *p* < 0.05).

### Dual-luciferase reporter assay

The canine *KLF4* and *VEGFA* 3’UTR clones, which include wild-type (WT) or mutated (MUT) miR-34a seed binding sites were cloned into the *PmeI* and *Xbal* sites of pmirGLO dual-luciferase miRNA target expression vector (Promega, Madison, WI, USA). OSA8 cells were cultured in 96-well plates in 75 μL RPMI medium supplemented with 10% heat-inactivated fetal bovine serum, without antibiotics. Cells were co-transfected at 80% confluency with 10 pmol control or pre-miR-34a mimic (Systems Biosciences) and 100 ng of WT or MUT *KLF4* or *VEGFA* pmirGLO constructs using Lipofectamine (Invitrogen). Cells were cultured for 24 h and luciferase activity was measured using a Dual-Glo® Luciferase Assay System (Promega) according to the manufacturer’s protocol. The luminescence of firefly luciferase substrate was normalized against that of *Renilla* luciferase substrate. For each transfection, the luciferase activity was averaged from three replicates. Sequences of WT and MUT *KLF4* 3’UTR were as follows: WT-5'-TAGTCCAAACAGTGGATGTGACCCA*CACTGCCA*GAAGAGAATTCAGTATTTTTT-3'; MUT -5'-TAGTCCAAACAGTGGATGTGACCCA*ACTGATTG*GAAGAGAATTCAGTATTTTTT-3'. Sequences of WT and MUT *VEGFA* 3’UTR were as follows: WT-5'-TAGTACGGGCATCTTGCCCCCAGGGG*CACTGCC*TGGAAGATTCAGGAGACTGGGCAGCCTTCACCTACTCT-3'; MUT- 5'-TAGTACGGGCATCTTGCCCCCAGGGG*AGTGAAT*TGGAAGATTCAGGAGACTGGGCAGCCTTCACCTACTCT-3’.

### Statistics

Experiments were performed in triplicate and repeated 3 times. Data were presented as mean plus or minus standard deviation. RT-qPCR miRNA or gene expression data was first normalized to internal control (snU6 and GAPDH, respectively) and the ΔΔCt method was used to compare miRNA expression by one-way analysis of variance (ANOVA) followed by Tukey-Kramer *post-hoc* test [48]. For analysis of all cell proliferation, cell cycle analysis, invasion, luciferase reporter assay, and RT-qPCR expression data, a linear mixed effects model was used to take account of the correlations among observations run in the same biological replicate. The difference between two group means was analyzed using the Student’s *t*-test and multiple group comparisons were analyzed by one-way analysis of variance (ANOVA) followed by Tukey-Kramer *post-hoc* test. *P*-values of less than 0.05 were considered statistically significant.

## Results

### MiR-34a expression is decreased in primary canine OSA tumors and canine OSA cell lines

**Fig 1:**
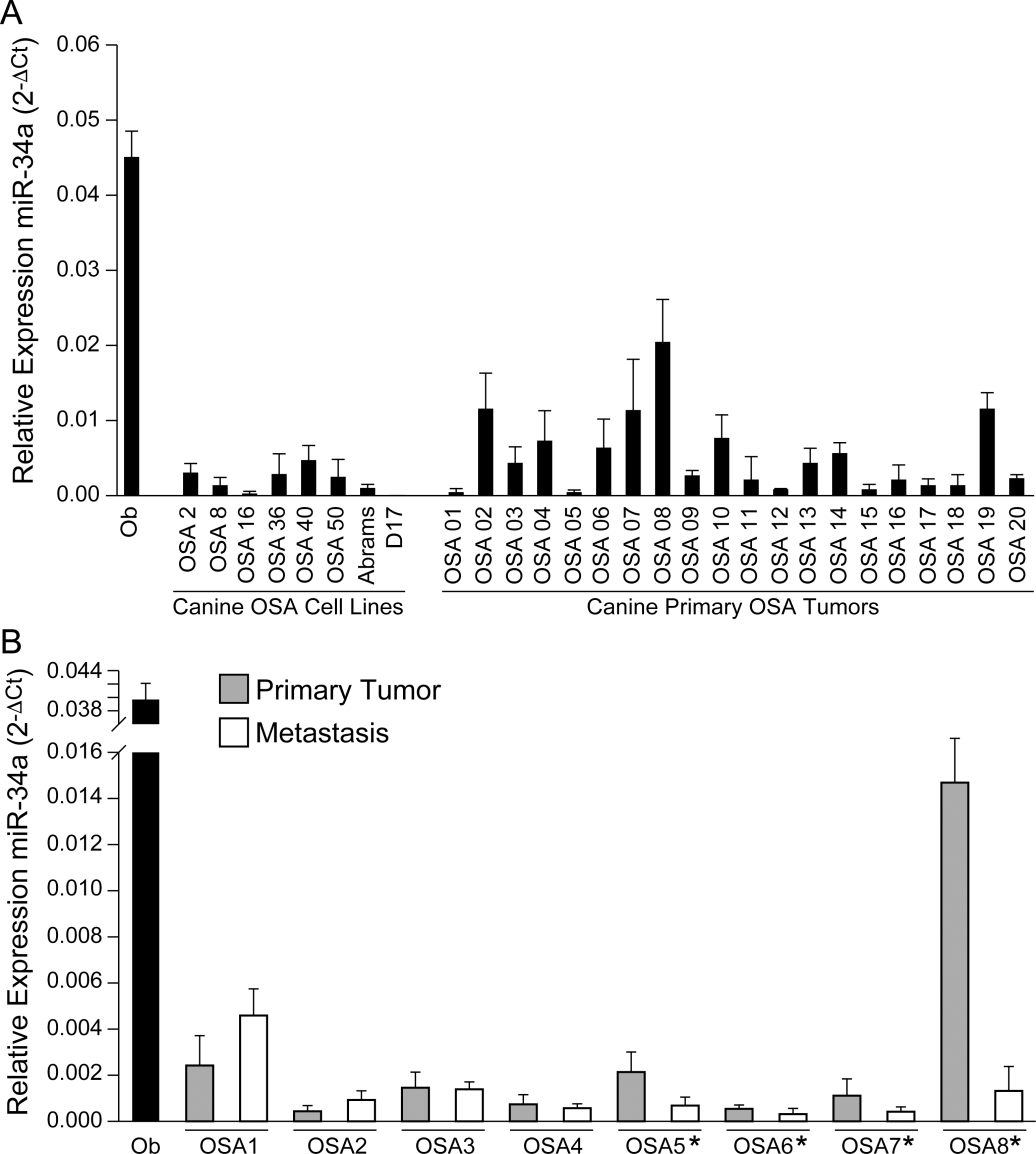
MiR-34a expression is reduced in primary canine OSA tumors and OSA cell lines. (A) RT-qPCR was used to assess mature miR-34a levels in primary canine OSA tumors (N = 20) and canine OSA cell lines (N = 8) relative to normal canine osteoblasts. Total RNA was reverse-transcribed and human Taqman miRNA assays were used to detect miR-34a expression. All results were normalized to snU6 control. All reactions were performed in triplicate and each experiment was repeated 3 times. Raw data were log transformed to reduce variance and skewness. Linear mixed effects models were applied to expression data to account for the correlation of the observations from the same batch. RT-qPCR results showed significant down-regulation of miR-34a in canine OSA tumors (*p* < 0.01) and OSA cells lines (*p* < 0.05) as compared to normal canine osteoblasts. Multiple group comparisons were analyzed by one-way ANOVA followed by Tukey-Kramer *post-hoc* comparison. (B) Metastasis-associated changes in miR-34a expression in canine OSA patients using paired primary OSA tumor biopsy and metastatic tumor tissue. MiR-34a expression was evaluated in paired primary canine OSA tumor biopsy and lung metastasis from 8 patients using Human Taqman miRNA assays to detect miR-34a as described previously. All results were normalized to snU6 control and data are shown relative to normal canine osteoblasts. While 2/8 samples showed increased miR-34a expression in metastatic tissues compared to primary tumor biopsies, 4/8 (50%) had decreased miR-34a expression; miR-34a levels were unchanged in 2 patients. Comparison between primary and metastatic tissues with a linear mixed models showed a 1.5-fold (0.9–2.3, 95% confidence interval) decreased expression of miR-34a in metastatic tissues compared to primary OSA tissues (*p*-value = 0.12).

We previously reported a miRNA expression signature that was associated with canine OSA using the nanoString nCounter platform, including down-regulation of miR-34a in primary canine OSA tumors. Concordant with these data, RT-qPCR confirmed that miR-34a expression is substantially decreased in canine OSA tumors as compared to normal canine osteoblasts (Fig 1). Although levels of miR-34a expression varied among OSA tumor samples, this may be attributed, in part, to heterogeneity in tumor stroma and/or inflammatory cells present in the tumor microenvironment. Furthermore, in a panel of canine OSA cell lines, miR-34a expression was found to be significantly decreased in all of the malignant OSA cell lines compared to that observed in normal canine osteoblasts. These findings are consistent with published data demonstrating that miR-34a expression levels are significantly decreased in human OSA tumor tissues and OSA cell lines [30, 42].

### MiR-34a expression is decreased in paired canine OSA metastatic lesions compared to primary OSA tumors

Given the highly metastatic behavior of OSA and data demonstrating that miR-34a influences OSA metastasis *in vivo* [42], miR-34a expression levels were evaluated in paired primary OSA tumors and metastatic lung lesions from eight canine patients. RT-qPCR showed that 50% (4/8) of paired specimens showed reduced miR-34a in metastasis compared to the primary tumor (Fig 1B). MiR-34a levels were unchanged in two of the paired samples (two already had reduced miR-34a levels in the primary tumor compared to normal osteoblasts), whereas two metastatic tissues exhibited higher miR-34a expression as compared to the primary OSA tumor biopsy. Therefore, down-regulation of miR-34a may be associated with the metastatic phenotype in canine OSA.

### Ectopic expression of miR-34a does not influence cell proliferation or cell cycle distribution in canine OSA cell lines

**Fig 2:**
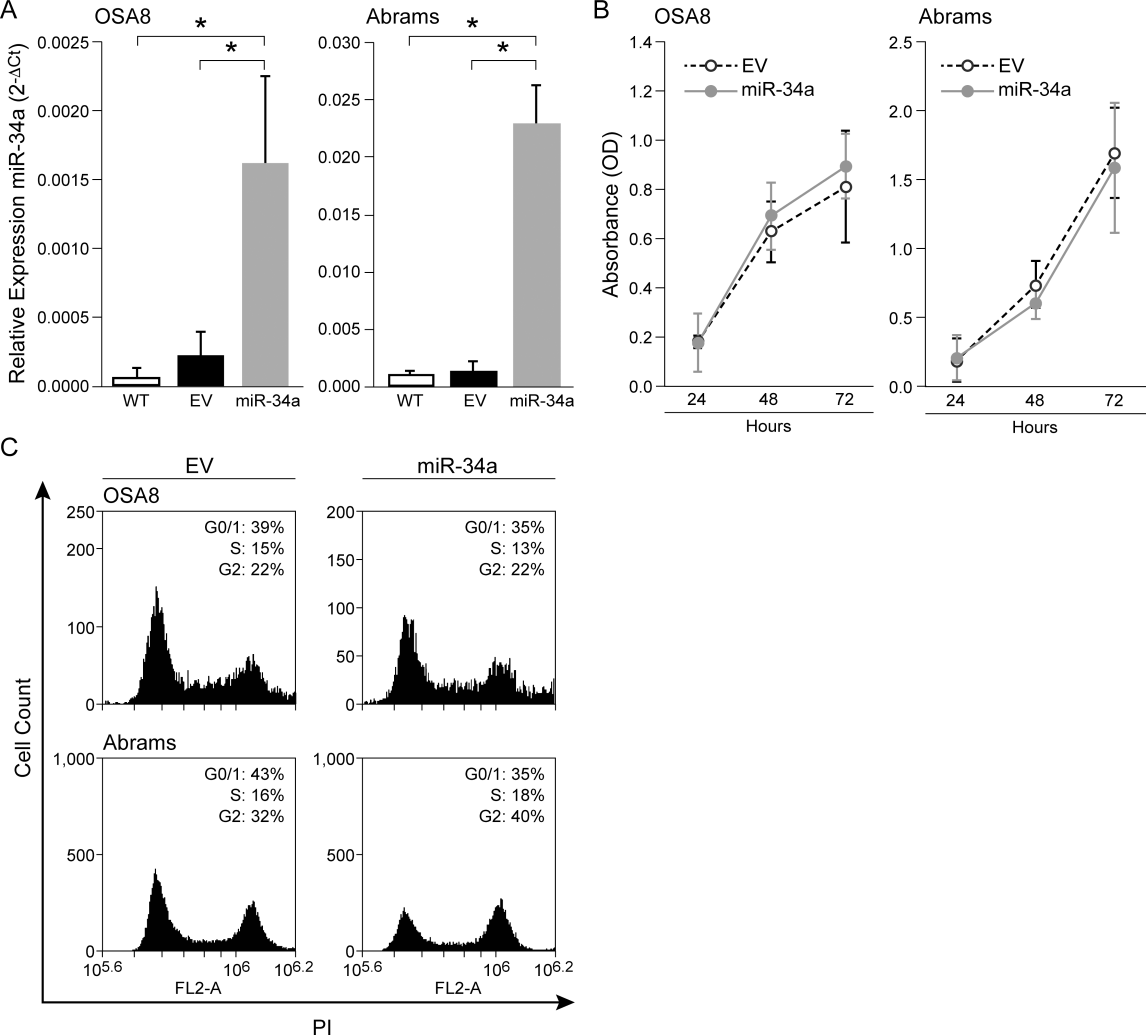
Ectopic expression of miR-34a in canine OSA cells does not affect cell proliferation or cell cycle distribution. (A) Canine OSA8 and Abrams cells were transduced with pre-miR-34a lentiviral constructs or empty control vector and sorted to greater than 95% purity based on GFP expression 72 hours following infection. Total RNA was isolated and RT-qPCR was performed as described above immediately prior to plating cells to confirm transduction efficiency miR-34a levels in wild-type (WT), empty vector (EV), and miR-34a expressing cells (*p < 0.05). Multiple group comparisons were analyzed by one-way ANOVA followed by Tukey-Kramer *post-hoc* comparison. (B) OSA8 and Abrams cells transduced with either empty control or pre-miR-34a lentiviral constructs were plated in complete media and the cell proliferation was assessed at 24, 48, and 72 hours using the BrdU incorporation assay. Cell proliferation was measured at 490 nm. Values of optical density (OD) are expressed as means +/- SD of 3 independent experiments. (C) OSA8 and Abrams cells expressing empty vector or pre-miR-34a lentivirus vector were incubated in complete media for 24 hours. Cells were then evaluated for effects on cell cycle using propidium iodide (PI) staining and flow cytometry. Three independent experiments were performed, and 1 representative result is presented. Linear mixed effects models were applied to OSA8 and Abrams cell line miR-34a expression, proliferation and cell cycling data to take account of the correlation among observations from the same replicates. No statistically significant difference in cell proliferation or cell cycle distribution was detected in OSA8 or Abrams cells expressing empty vector or pre-miR-34a vector for any of the tested time points (Student’s *t*-test).

To investigate the functional consequences of miR-34a expression on OSA cell behavior, we stably expressed miR-34a in the OSA8 and Abrams cells lines. OSA8 and Abrams cell lines were transduced with empty or pre-miR-34a expressing lentivirus vector and 72 hours post transduction, cells were sorted based on GFP-positivity and overexpression of miR-34a was confirmed by RT-qPCR (Fig 2A).

The impact of miR-34a overexpression on the proliferative capacity of OSA cells was assessed using a bromodeoxyuridine (BrdU) incorporation assay. No effects of miR-34a expression on proliferation of the OSA8 or Abrams cell line were observed at multiple time points when compared to cells expressing empty vector (Fig 2B). Concordant with this finding, miR-34a expression did not alter cell cycle status in either the OSA8 or Abrams cells as assessed by propidium iodide (PI) staining (Fig 2C).

### MiR-34a expression decreases cell invasion and migration in canine osteosarcoma cell lines

**Fig 3:**
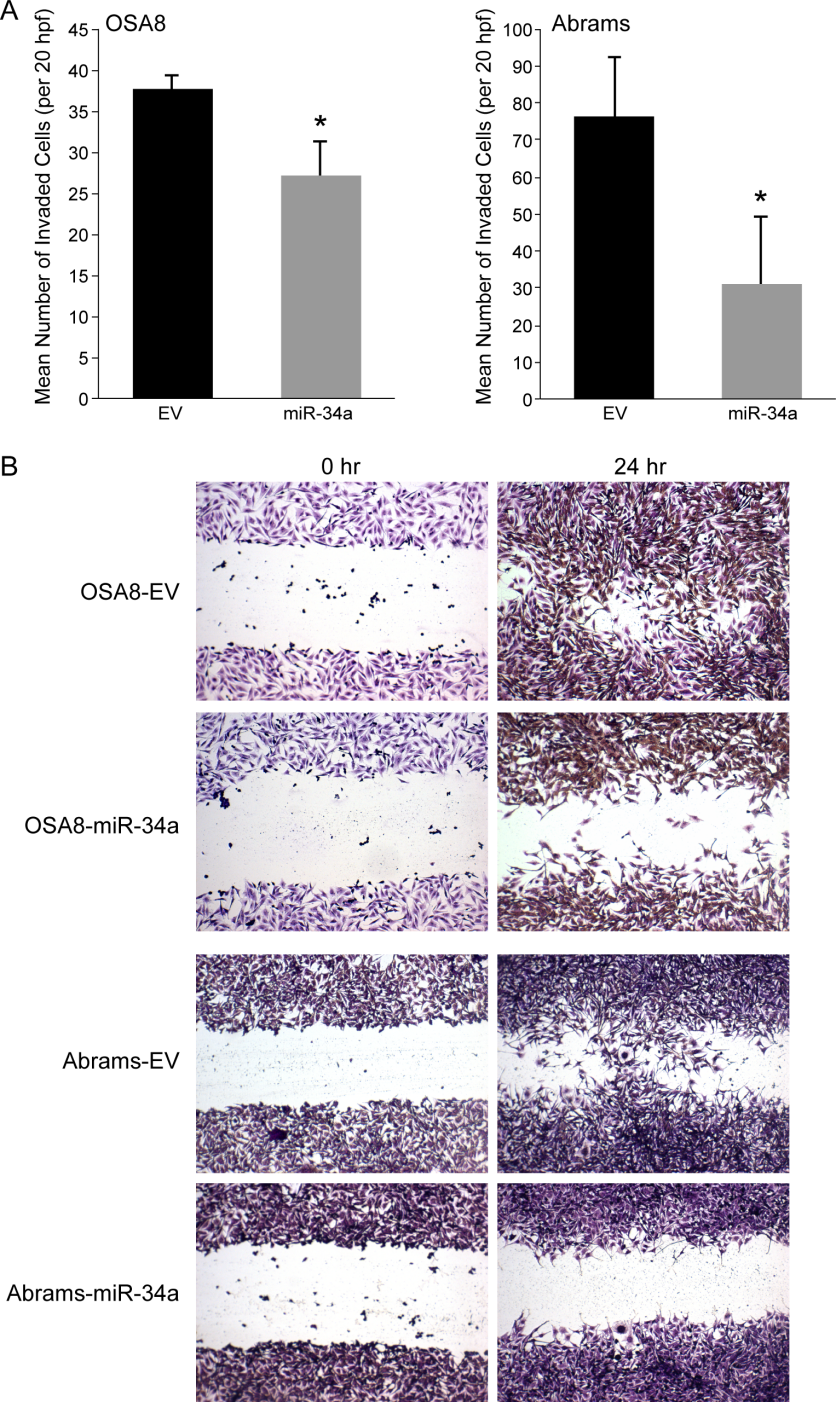
Expression of miR-34a in canine OSA cell lines suppresses cell invasion and migration. (A) Canine OSA8 and Abrams cells expressing control or pre-miR-34a lentiviral constructs were plated in serum free media in the upper wells of plates for Matrigel invasion assays. The lower chamber of each well contained complete media supplemented with 10% fetal bovine serum. Cells were allowed to invade for 24 hours through a layer of Matrigel basement membrane before removal of Matrigel and media from the upper chambers. Cells that had migrated to the lower surface of the insert membrane were stained with crystal violet and the number of invaded cells were counted in ten random fields in triplicate replicates. Experiments were performed in triplicate and data are represented as the mean ± standard deviation (*p ≤ 0.05, Student’s *t*-test). (B) Migration behavior was assessed in OSA8 and Abrams cell lines expressing empty vector or pre-miR-34a lentiviral constructs using standard wound-healing assays. Cells were seeded in 6-well plates in complete growth medium and allowed to grow to 70% confluency. A scratch was introduced using a P200 pipet tip and after 24 hours, cells were fixed, stained with crystal violet and evaluated by digital photography.

Prior studies indicated that miR-34a expression alters the invasive capacity of tumor cells [42]. Similarly, overexpression of miR-34a significantly inhibited cell invasion through Matrigel in the OSA8 and Abrams cells as compared to cells expressing empty vector (Fig 3A). Furthermore, miR-34a decreased cell motility and scattering as compared to control cells in both cell canine OSA cell lines (Fig 3B). Taken together, these findings demonstrate that miR-34a inhibits the invasive capacity and migratory behavior of canine OSA cell lines.

### RNA sequencing identifies miR-34a-induced gene alterations in canine OSA8 cells

**Fig 4:**
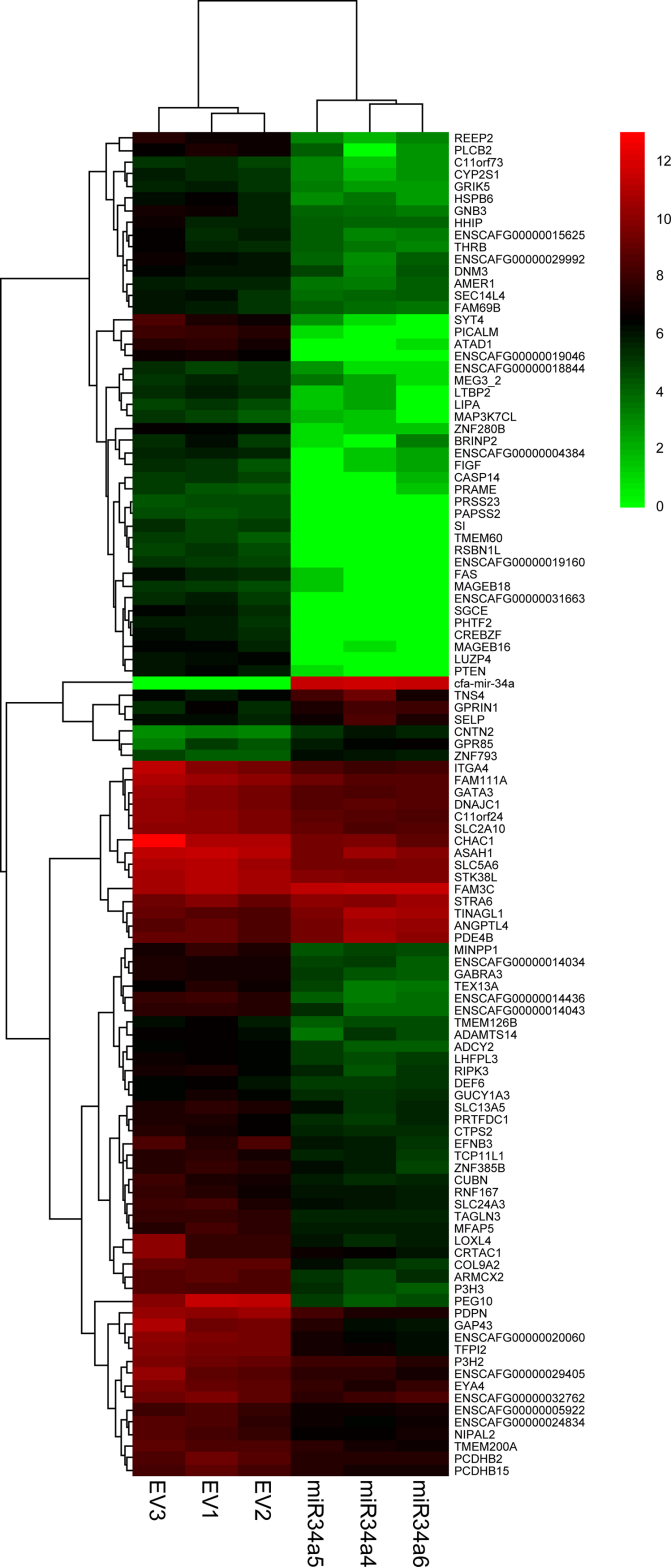
Overexpression of miR-34a in canine OSA8 cells significantly alters gene expression. Total RNA was isolated from canine OSA8 cells expressing control or pre-miR-34a lentiviral constructs from three separate transduction experiments and next-generation sequencing was performed to identify differences in gene transcript expression. Supervised hierarchical cluster analysis of 111 genes differentially expressed in OSA8 cells expressing either empty vector (EV) or miR-34a (miR34a) as determined by one-way ANOVA comparison test (*p* ≤ 0.0001). Color areas indicate relative expression of each gene after log2 transformation with respect to the gene median expression (red and green colors denote high and low expression, respectively).

To understand the molecular mechanisms underlying the miR-34a associated decrease in cell motility and invasion in canine OSA cell lines, RNA sequencing was performed on the OSA8 cells expressing either control or pre-miR-34a lentiviral constructs. Overexpression of miR-34a significantly altered the gene expression profile of OSA8 cells, including substantial up-regulation (fold change ≥ 1.5) of 12 genes and downregulation (fold change ≤ -1.5) of 99 gene transcripts when compared to control OSA8 cells (Fig 4, *p* ≤ 0.0001; S2 Table).

Computer-aided bioinformatics algorithms (TargetScan, miRanda. MiRWalk, miRDB) were used to identify predicted miR-34a target genes containing putative miR-34a binding sites within their 3’-UTR. We identified 150 genes containing consensus binding sites for miR-34a that were significantly down-regulated (fold change ≤ -1.4, *p*-value < 0.05) following enforced expression of miR-34a in OSA8 cells, demonstrating that the miR-34a-downregulated genes are enriched for direct targets of this miRNA (S3 Table). Furthermore, we analyzed the Gene Ontology (GO) classifications of these genes using ToppGene Suite (http://toppgene.cchmc.org) and found that putative miR-34a target genes are highly enriched for biological processes related to cell migration, including GO categories “regulation of cell projection organization” (*p*-value 7.55 x 10^−5^), “regulation of locomotion” (*p*-value 1.96 x 10^−4^), and “cell projection organization” (*p*-value 3.90 x 10^−4^), consistent with the role of miR-34a in mediating cellular invasion and motility in OSA cells (S4 Table).

**Fig 5:**
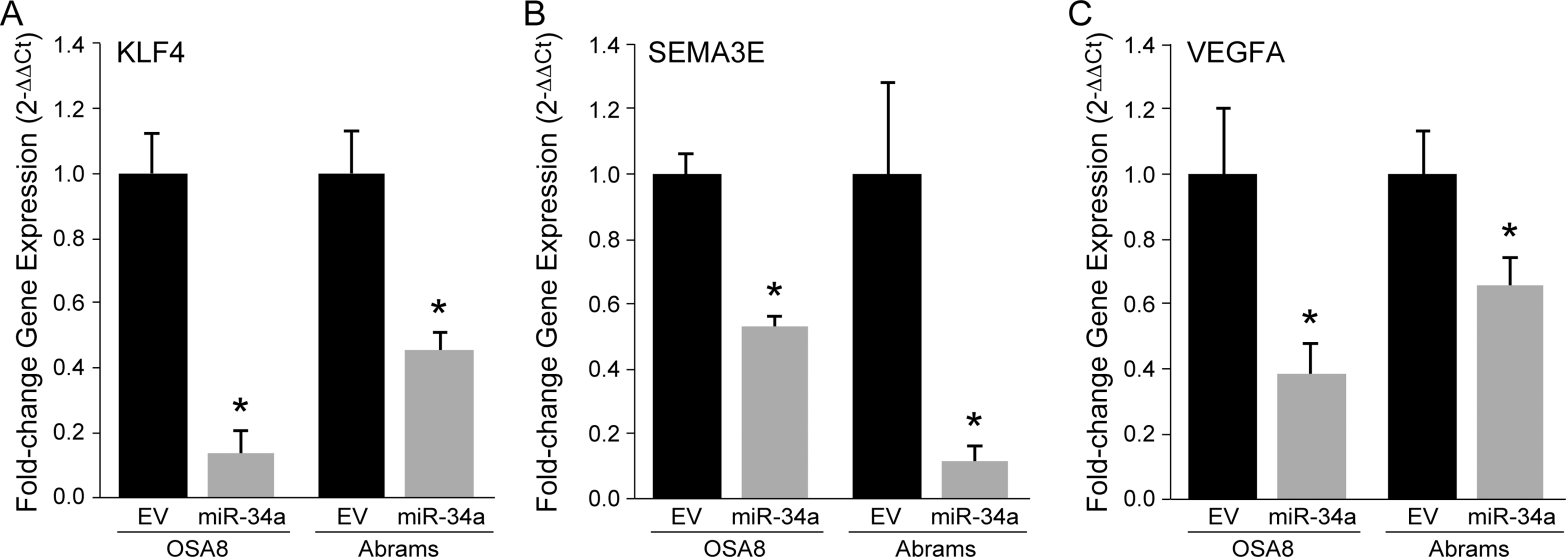
Identification of putative miR-34a target genes dysregulated by miR-34a overexpression in canine OSA cell lines. Transcriptional profiling of canine OSA8 cells expressing pre-miR-34a (miR-34a) or empty vector (EV) control was performed by next-generation sequencing to identify genes showing differential expression with miR-34a overexpression. RT-qPCR was performed to independently validate changes in gene expression for putative miR-34a targets (A) KLF4, (B) SEM3AE, and (C) VEGFA altered by miR-34a overexpression in both canine OSA8 and Abrams cell lines. Data presented show the mean fold change (relative to GAPDH control) ± standard deviation (Student’s *t*-test, *p < 0.01).

We identified putative miR-34a target genes that were common to all three GO categories related to cell migration including Krüppel-like factor 4 (KLF4), Semaphorin 3E (SEMA3E), and Vascular endothelial growth factor A (VEGFA). RT-qPCR was performed on OSA8 cells expressing empty vector or miR-34a to validate changes in mRNA expression following miR-34a overexpression. Consistent with our RNA sequencing results, transcripts for KLF4, SEMA3E, and VEGFA were down-regulated following overexpression of miR-34a in OSA8 cells, suggesting that miR-34a may alter the invasive capacity and migratory behavior of OSA8 cells, in part, by regulating the expression of these genes (Fig 5). To investigate whether similar alterations in transcript expression occurred following overexpression of miR-34a in other canine OSA cell lines, RT-qPCR was performed for KLF4, SEMA3E, and VEGFA in the canine Abrams OSA cell line expressing either empty control or miR-34a lentivectors (Fig 5). In agreement with our findings in the OSA8 cell line, transcript levels for KLF4, SEMA3E, and VEGFA were substantially decreased following enforced miR-34a expression in the Abrams cell line.

### MiR-34a directly targets KLF4 and VEGFA in canine OSA cells

**Fig 6:**
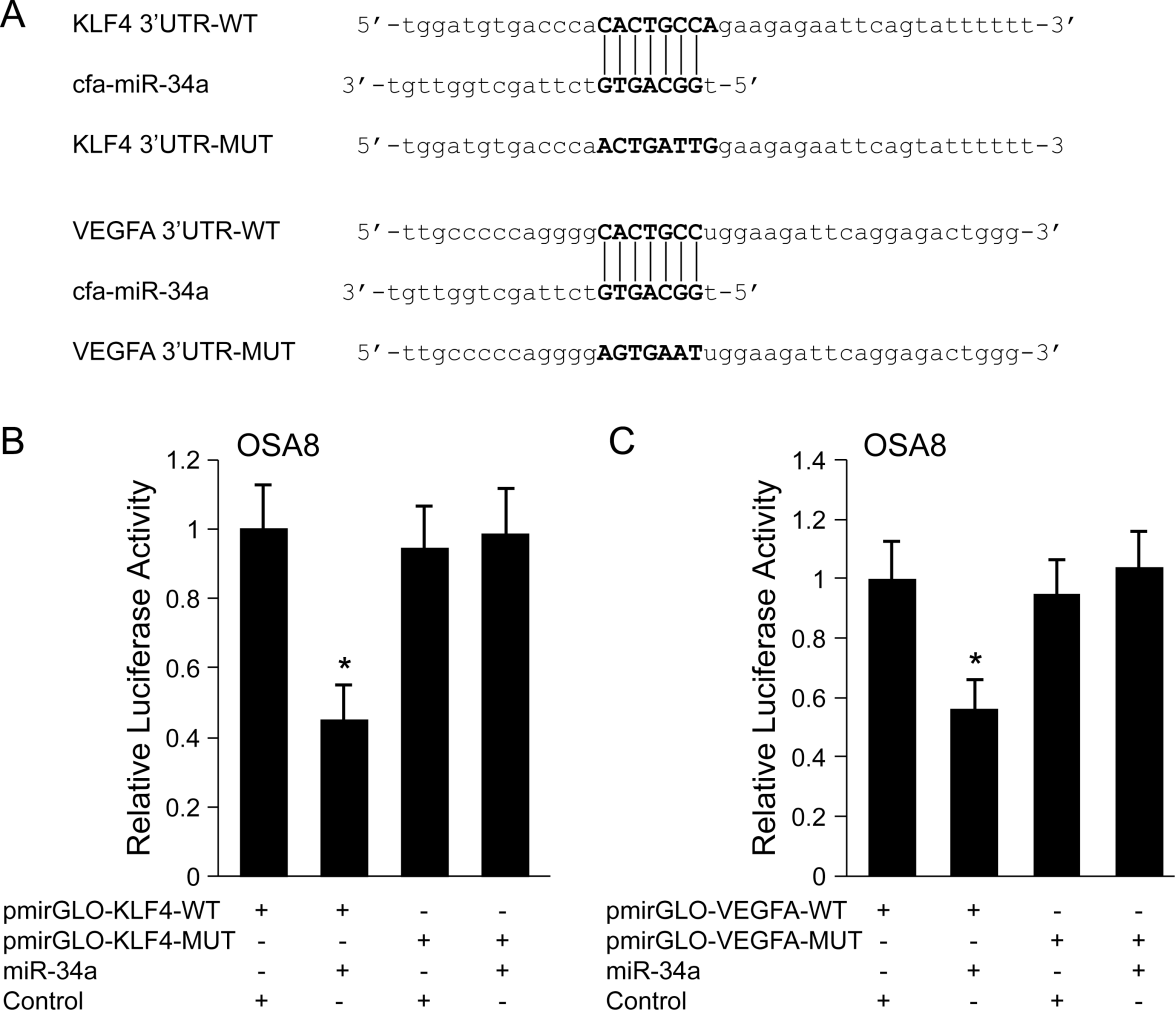
KLF4 and VEGFA are direct targets of miR-34a. (A) Predicted miR-34a binding sites in the 3’UTR of canine KLF4 and VEGFA. Reporter constructs harboring wild-type (WT) or seed sequence-mutated (MUT) miR-34a binding sites for canine KLF4 and VEGFA were cloned into the pmirGLO Dual-Luciferase miRNA Target Expression Vector. (B, C) Canine OSA8 cells were co-transfected with control or miR-34a mimics and pmirGLO vectors, possessing either the WT or MUT 3’UTR sequence for KLF4 or VEGFA. Cells were harvested after 24 h and assayed for dual luciferase activity. Luciferase activity was normalized to *Renilla* control reporter activity. Data shown are means ± standard deviation from a representative of three independent experiments (one-way ANOVA followed by Tukey-Kramer *post-hoc* test, *p < 0.05).

To determine whether putative miR-34a target genes identified by *in silico* computer-based predictive algorithms are directly regulated by miR-34a, the 3’-UTR of canine KLF4 and VEGFA containing wild-type (WT) or mutant (MUT) miR-34a target sequences were cloned downstream of a luciferase reporter gene in the pmirGLO Dual-Luciferase miRNA Target Expression Vector (Fig 6A). Canine OSA8 cells were co-transfected with miR-34a or control vector and pmirGLO constructs harboring the WT or MUT 3’UTRs of KLF4 or VEGFA and luciferase activity was evaluated. The results of the dual-luciferase assay showed that miR-34a significantly suppressed the luciferase reporter activity of pmirGLO-KLF4-3’UTR-WT and pmirGLO-VEGFA-3’UTR-WT (Fig 6B and 6C). In contrast, this was almost completely abrogated upon transfection of reporter plasmids containing mutant 3’UTRs (pmirGLO-KLF4-3’UTR-MUT or pmirGLO-VEGFA-3’UTR-MUT), indicating that miR-34a negatively regulates KLF4 and VEGFA in OSA8 cells via direct binding to the 3’-UTR of these transcripts.

### Putative miR-34a target gene expression is increased in primary canine OSA tumors

**Fig 7:**
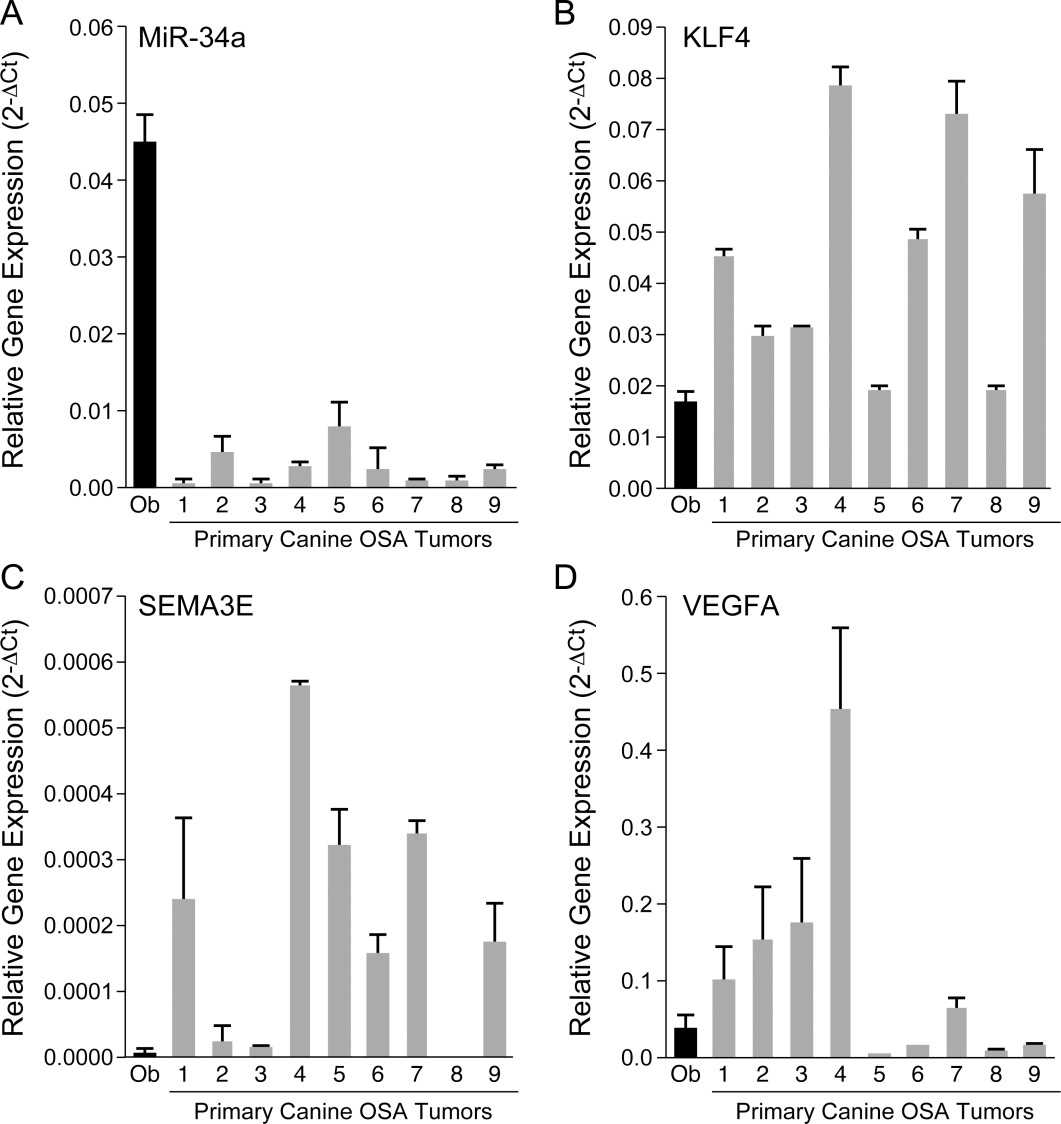
Evaluation of putative miR-34a target expression in primary canine OSA tumors. (A) RT-qPCR evaluating mature miR-34a expression in primary canine OSA tumors (N = 9) and normal canine osteoblasts demonstrated that the mean expression of miR-34a was significantly reduced in OSA tumors compared to osteoblasts (Bars: SD. Statistical analysis: one-way ANOVA followed by Tukey-Kramer *post-hoc* comparison, *p < 0.05). All reactions were performed in triplicate and results were normalized to snU6. Transcript levels of putative miR-34a target genes (B) KLF4, (C) SEM3AE, and (D) VEGFA were assessed in primary canine OSA tumors and normal canine osteoblasts using RT-qPCR. All reactions were performed in triplicate and results were normalized to GAPDH. Raw data were log transformed to reduce variance and skewness. Linear mixed effects models were applied to expression data to account for the correlation of the observations from the same batch.

To determine whether expression levels of putative miR-34a target genes are increased in spontaneous canine OSA tumors, we performed RT-qPCR to evaluate gene transcript expression (KLF4, SEMA3E, and VEGFA) in primary canine OSA tumors and normal canine osteoblast cells. We first confirmed that basal levels of miR-34a were significantly lower in the primary canine OSA tumors as compared to normal canine osteoblasts (Fig 7A). As shown in Fig 7, RT-qPCR demonstrated that primary canine OSA tumor tissues express high levels of putative miR-34a target genes, KLF4 and SEMA3E. In contrast, normal canine osteoblasts possessing high basal levels of miR-34a expressed significantly lower levels of KLF4 and SEMA3E compared to OSA tumor samples. Interestingly, transcript levels of VEGFA varied among tumor samples and differential expression of VEGFA was not statistically significant in OSA tumors compared to osteoblast cells; however, this may have been due, in part, to variations in stroma/inflammatory cells within the tumor microenvironment or baseline necrosis within the primary tumor specimen that influenced the proportion of tumor cells. Taken together, these findings support the assertion that loss of miR-34a may promote a pattern of gene expression contributing to the metastatic phenotype in canine OSA.

## Discussion

The contribution of miRNAs to cancer has been widely confirmed by numerous studies demonstrating aberrant expression of miRNAs in almost all types of human cancer. The miR-34 family, most notably miR-34a, is frequently lost or down-regulated in human malignancies including neuroblastoma, breast, lung, and colorectal carcinomas, and osteosarcoma. Furthermore, the introduction of miR-34a gene constructs into cell lines derived from solid tumors (lung, liver, colon, pancreatic, brain, prostate, bone, ovary) and hematopoietic malignancies (lymphoma, leukemias) induces apoptosis and cell cycle arrest, and inhibits migration and invasion, providing support for a tumor suppressor function of miR-34a [25, 26, 40]. Given the prevalence of miR-34a dysregulation in cancer and its ability to down-regulate the expression of numerous oncogenes across multiple oncogenic pathways, there is ongoing interest in developing miR-34a mimic therapy as a novel therapeutic strategy for cancer. Indeed, a variety of miRNA formulations and target-specific delivery strategies have accelerated the clinical development of miR-34 mimics, Miravirsen (Santaris Pharma) and MRX34 (Mirna Therapeutics) which recently entered first-in-human phase I clinical trials (NCT01829971) in patients with advanced solid tumors [50–52].

With respect to the potential role of miR-34a dysregulation in osteosarcoma (OSA), several studies have demonstrated decreased expression in human OSA. Importantly, miR-34a loss in OSA is associated with enhanced expression of a several targets known to contribute to tumorigenesis including MET, SIRT1, and CDK6 [42, 53, 54] although its contribution to the biology of OSA has not yet been fully elucidated. In our prior work we evaluated miRNA expression signatures in canine OSA and found that canine OSA tumors similarly express low levels of miR-34a. Given that canine OSA is a well validated spontaneous large animal model of the human disease, the purpose of this study was to characterize the impact of miR-34a expression in canine OSA tumor lines to better understand its potential role in the biology of this disease.

Our data demonstrate that expression of miR-34a is significantly decreased in primary canine OSA tumor tissues and cell lines compared to normal canine osteoblasts. Although variability in miR-34a expression levels was observed among primary OSA tumor samples, this may be attributed, in part, to heterogeneity in tumor stroma and/or non-neoplastic cell infiltrates present in the tumor microenvironment. Our finding that miR-34a expression is decreased in canine OSA is consistent with previous studies demonstrating that miR-34a expression is reduced or absent in human OSA tumor samples compared to paired non-cancerous bone and suggests that loss of miR-34a is a common event in both species [30]. We analyzed miR-34a expression levels in primary canine OSA tumors and paired metastases and found that half of metastatic lesions had reduced levels of miR-34a compared with their primary tumor. The limited number of paired tumor samples evaluated in the current study precludes us from drawing conclusions about the expression of miR-34a in OSA metastases. Nevertheless, the finding that miR-34a levels are decreased in a subset of metastatic OSA tumors merits further investigation as to the role of miR-34a in promoting the metastatic phenotype.

Studies have shown that ectopic expression of miR-34a through various methods such as chemically modified oligonucleotide miR-34a mimics and bioengineered miR-34a prodrugs results in decreased viability, migration and invasion of human OSA cell lines [43, 53, 54]. In the canine OSA cell lines, stable overexpression of miR-34a resulted in significant inhibition of cellular invasion and migratory capacity. Interestingly, enforced expression of miR-34a had no effect on proliferation or cell cycle distribution in the cell lines evaluated. However, this may be explained by differences in the expression of mRNA targets in distinct cell types/tissues that influence the effect of miR-34a on cell behavior. Similarly, differences in the capacity of miR-34a to inhibit cell viability or suppress cellular migration and invasion have been observed following miR-34a overexpression in different human OSA tumor cell lines [42, 43]. These cross-species data support the notion that miR-34a plays a role in regulating the invasive capacity of OSA cells.

Enforced expression of miR-34a decreases the invasive capacity of human OSA cell lines *in vitro* and modulates the progression of OSA lung metastasis *in vivo*, in part, through direct targeting of oncogenes such as c-Met, sirtuin 1 (SIRT1), and CD44 [42, 43, 54]. However, given the broad capacity for miRNAs to regulate multiple genes and it is likely that miR-34a affects a range of genes that participate in metastasis [14]. To begin to dissect the underlying mechanisms mediating the observed miR-34a-dependent effects in canine OSA cells, RNA sequencing was performed on canine OSA8 cells expressing control or miR-34a lentiviral constructs. Notably, enforced expression of miR-34a significantly altered the transcriptome of canine OSA8 cells and 150 of 773 down-regulated genes (19%) contained consensus binding sites for miR-34a, demonstrating enrichment for direct targets of this miRNA. Furthermore, gene ontology analysis of these miR-34a target genes demonstrated associations with biological processes related to cell migration. We validated the expression profiles of select putative miR-34a target genes including Krüppel-like factor 4 (KLF4), Semaphorin 3E (SEMA3E), and Vascular endothelial growth factor receptor A (VEGFA) and confirmed significant down-regulation of these genes in OSA8 and Abrams cells overexpressing miR-34a. Lastly, we confirmed direct targeting of KLF4 and VEGFA transcript 3’UTR by miR-34a, suggesting a potential mechanism by which miR-34a inhibits invasion and migration in canine OSA cells. Semaphorins comprise a family of secreted and membrane-associated proteins that play multifunctional roles in embryonic development, immune regulation, angiogenesis, cancer, and bone formation [55, 56]. Accumulating evidence suggests that signaling cascades triggered by class 3 semaphorins play a crucial role in regulating bone homeostasis, osteoblast differentiation and osteodeposition and their dysregulation is implicated in malignant bone pathology. In support of this idea, SEMA4D and SEMA6D transcripts were found to be highly expressed in human OSA tumors compared to normal osteoblasts and over 50% of tumors on an OSA tissue microarray were shown to express high levels of these proteins [57]. While the function of Sema3E has not been evaluated in osteosarcoma, the importance of Sema3E in promoting the metastatic phenotype has been explored in murine mammary adenocarcinoma cells, which show enhanced cellular invasion and rapid experimental lung metastasis formation *in vivo* in response to Sema3E overexpression [58, 59]. Moreover, the expression of Sema3E in human tumor samples correlates with metastatic progression and in support of these findings, knockdown of Sema3E in human carcinoma cell lines reduced their metastatic potential when injected into mice [60–62].

Krüppel-like factor 4 (KLF4) is a zinc-finger transcription factor that is often overexpressed in human primary OSA tumors compared normal bone and is highly expressed in OSA stem cell populations [63, 64]. ShRNA-mediated knockdown of KLF4 in human MG63 and SaOS2 cell lines inhibited cell clonogenicity and abrogated cell migration in wound-healing and Matrigel migration assays and these effects were directly related to downregulation of the KLF4 transcriptional target, CRYAB [63]. Furthermore, inhibition of KLF4 in human OSA xenografts reduced tumor growth *in vivo*, providing further support for its role in OSA biology.

During the process of hematogenous metastasis, VEGFA is suggested to play a major role in regulating angiogenesis. Ectopic expression of miR-34a in human head and neck squamous cell carcinoma (HNSCC) cells was found to inhibit tumor growth and angiogenesis that was partially reversed by blocking VEGFA production by tumor cells [65]. Concordant with these data, stable overexpression of miR-34a in canine OSA cell lines reduced VEGFA expression with a concomitant decrease in cell invasion and migration. Higher levels of VEGFA expression in human OSA tumors have been shown to correlate with the presence of lung metastasis and VEGFA levels have predictive value for survival of OSA patients [66]. With respect to the canine disease counterpart, higher levels of plasma VEGFA were found in more aggressive neoplasms in a survey of spontaneously occurring tumors in dogs, including OSA [67]. Furthermore, activation of ΔNp63 has been shown to promote cellular invasion, migration and angiogenesis of canine OSA cell lines, in part through its effects on VEGFA, IL-8, and STAT3 phosphorylation [68]. Collectively, these data support the notion that enhanced VEGFA production in canine OSA cells may promote angiogenesis, thereby contributing to the metastatic cascade.

Concordant with the observation that miR-34a decreased the expression of SEM3A, KLF4, and VEGFA in canine OSA cell lines and that miR-34a negatively regulates KLF4 and VEGFA transcripts, we found that in primary canine OSA tumors expressing low basal levels of miR-34a, expression of these putative target genes was significantly increased compared to normal osteoblasts possessing high miR-34a levels. These data provide a further association between miR-34a and regulation of cellular pathways that promote a metastatic phenotype. Loss and gain of function studies would be required to further elucidate the contribution of these targets to normal and malignant osteoblast invasion and as such, this represents an ongoing area of investigation.

## Conclusions

Our data demonstrate that miR-34a expression is significantly decreased in primary canine OSA tumor tissues and canine OSA cell lines compared to normal canine osteoblasts. Our findings are concordant with data generated in human OSA tumors, suggesting that loss of miR-34a may be common in this disease. Enforced expression of miR-34a in canine OSA cell lines reduced cellular invasion and migration and significantly altered the transcriptional profile of OSA cells, including down-regulation of several genes implicated in promoting the metastatic phenotype. This work serves as the foundation for future work to dissect the molecular mechanisms by which miR-34a regulates the invasive capacity of canine OSA cells, with the ultimate goal of identifying new targets for therapeutic intervention in this disease.

## Supporting information

S1 Table Clinical patient data. Clinical patient data including age, sex, breed, histopathological diagnosis, and primary tumor location.
(XLS)

S2 Table Gene transcripts altered by miR-34a overexpression in canine OSA8 cells. Gene transcripts differentially expression in canine OSA8 cells expressing empty vector (EV) or miR-34a as determined by one-way ANOVA comparison test (*p* < 0.0001).
(XLSM)

S3 Table Predicted miR-34a target genes down-regulated with miR-34a expression in canine OSA8 cells. Computer-aided bioinformatics algorithms (TargetScan, miRanda. MiRWalk, miRDB) were used to identify predicted miR-34a target genes containing putative miR-34a binding sites within their 3’-UTR. 150 genes that were significantly down-regulated (fold change ≤ -1.4, *p*-value < 0.05) following enforced expression of miR-34a in OSA8 cells were found to contain consensus binding sites for miR-34a using *in silico* miRNA prediction tools.
(XLSX)

S4 Table Gene ontology classification of predicted miR-34a target genes. ToppGene Suite (http://toppgene.cchmc.org) was used to analyze Gene Ontology (GO) classifications of predicted miR-34a target genes.
(XLSX)
